# Active Motor Training Has Long-term Effects on Infants’ Object Exploration

**DOI:** 10.3389/fpsyg.2016.00599

**Published:** 2016-05-02

**Authors:** Sarah E. Wiesen, Rachel M. Watkins, Amy Work Needham

**Affiliations:** ^1^Infant Learning Lab, Department of Psychology and Human Development, Peabody College, Vanderbilt University, NashvilleTN, USA; ^2^Auditory Development Lab, Department of Hearing and Speech Sciences, Vanderbilt University, NashvilleTN, USA

**Keywords:** infancy, motor development, early motor intervention, grasping, sticky mittens, object exploration

## Abstract

Long-term changes in infants’ behavior as a result of active motor training were studied. Thirty-two infants completed three visits to the laboratory. At the first visit, infants were 3 months old and completed an object exploration assessment. Then the experimenter demonstrated the motor training procedures appropriate for the infant’s experimental condition, and parents took home custom infant mittens (either sticky or non-sticky) and a bag of lightweight toys to practice with their infants. Over the course of the following 2 weeks, infants participated in 10 sessions of either active (sticky) or passive (non-sticky) mittens training at home with their parents. Infants who participated in active mittens training wore mittens with the palms covered in Velcro, allowing them to pick up and move around small toys. Infants who participated in passive mittens training wore non-sticky mittens, and their parents moved the toys through their visual fields on their behalf. After completing the training, infants returned to the lab for the second visit. At visit two, infants participated in another object exploration assessment as well as a reaching assessment. Parents returned the training materials to the lab at the second visit, and were told not to continue any specific training regimen from this point forward. Two months later, when infants were about 5.5 months of age, they returned to the lab for a third visit. At the third visit, infants completed the same two assessments as during the second visit. The results of this study indicate that infants who participated in active motor training engaged in more sophisticated object exploration when compared to infants who received passive training. These findings are consistent with others in the literature showing that active motor training at 3 months of age facilitates the processes of object exploration and engagement. The current results and others reveal that the effects of early experience can last long after training ceases.

## Introduction

Many years ago, Held and Hein (1963) showed that visually inexperienced kittens learn to guide their actions more effectively after receiving active experience (which they controlled) than they did after receiving passive experience (which they did not control). These classic findings have led many researchers to consider the possibility that visual-motor experience that is controlled and observed by the same individual is critically important for effective learning about moving the body.

One possibility is that infants’ learning about how to reach independently is facilitated by observing their own successful actions upon objects. The transition into independent reaching has monumental consequences for infants. Before infants begin reaching, they spend a lot of time on their backs engaging in social interactions (Lobo and Galloway, 2008). Once they begin reaching, infants’ opportunities for learning about objects increase dramatically. They begin making purposeful actions on objects and can see the consequences of these action attempts.

Upon successfully contacting objects independently, infants can explore them. Through exploring objects, infants may learn about object properties, such as texture, color, shape, and weight, and about the effects of their own actions on objects (Gibson, 1988; Lederman and Klatzky, 2009). Learning about objects earlier can lead to language learning opportunities as infants learn about objects’ similarities, differences, and how objects relate to one another (Lifter and Bloom, 1989; Iverson, 2010; Lobo and Galloway, 2013). Object exploration may also allow infants to learn about object affordances, object categories, and how to use tools to achieve goals. Early object exploration might also influence infants’ problem solving abilities. Depth of exploration has been linked with infants’ problem solving abilities (Caruso, 1993), so enhancing infants’ exploration skills could also boost their problem solving skills.

One question of interest is what motivates infants to attempt to contact and explore objects in their surroundings. A suggested answer to this question, offered many years ago, is that young humans have a drive for competence, which includes understanding the physical objects in their environment (White, 1959; Hunt, 1965). More recently, Von Hofsten (2007) describes two sources of motivation that drive infants’ exploration. First, infants find novel objects and events intriguing. Secondly, infants are eager to exercise their new action abilities and find it rewarding to practice their new skills (Berger, 2010; Libertus and Needham, 2014). These factors motivate infants to make repeated efforts to influence their environments. So, manipulating infants’ exposure to novel toys or outcomes in response to their actions, or providing early opportunities for infants to practice their action skills may increase infants’ motivation to explore.

Prior research has established that 2 weeks of parent-led motor training leads infants to transition into reaching earlier and increases their object exploration skills (Needham et al., 2002; Heathcock et al., 2008; Libertus and Needham, 2010, 2011, 2014). Infants who participated in the active training condition of this early motor intervention began contacting objects and showing increased interest in exploring objects earlier than if they had instead participated in the passive training experience (Libertus and Needham, 2010, 2011, 2014). What features of early motor interventions have led to improvements in infants’ early reaching behaviors? Libertus and Needham (2014) examined the role of verbally encouraging infants to contact objects as well as the role of infants’ practice physically moving objects. They concluded that both parents’ verbal encouragement and practice moving objects themselves are key in promoting the earlier emergence of reaching. Heathcock et al. (2008) found that embedding early motor interventions into infants’ and families’ daily lives, by showing parents how to facilitate early motor interventions during daily activities with their infants, successfully eliminated the reaching delay that preterm babies are at risk for developing.

The current research utilized sticky mittens training, during which infants wear custom fleece mittens and interact with lightweight toys. In the active training condition, the palms of the mittens are covered in Velcro. Parents present infants with lightweight objects covered in Velcro strips. In the passive training condition, infants’ mittens are not covered in Velcro, and parents manipulate the toys on infants’ behalf to provide a similar visual experience. Past research found that infants who had 2 weeks of active mittens training showed more visually coordinated swatting motions, alternated between looking and mouthing more frequently, and began reaching earlier (Needham et al., 2002; Libertus and Needham, 2010).

Although prior research has not examined whether there is a lasting impact of mittens training on infants’ object exploration, new research has begun to address this question (Libertus et al., in press). In one study, infants who completed 2 weeks of either active or passive mittens training (as described above) at 3 months of age were re-tested 1 year later. The 15-month-old infants who participated in the active mittens training experience a year earlier showed more visual and manual engagement with a toy during a free exploration task than those who had received passive experience a year earlier (Libertus et al., in press).

These results suggest that the sticky mittens training intervention could set in motion a cascade of new learning opportunities; boosting infants’ object exploration skills could provide a strong foundation supporting infants’ future skills in multiple domains (Bornstein et al., 2013). However, we do not know whether infants’ object exploration behaviors show steady improvements throughout the first year of life as a result of active mittens training. Also, the tasks used in the Libertus et al. (in press) study were age appropriate for 15-month-old infants, but they were different from the object exploration tasks used in the original research.

In the current study, we sought to more fully understand the developmental trajectory of this process by probing the effects of active mittens training 2 months after infants concluded mittens training using the same object exploration assessment that has been used in previous research.

## Materials and Methods

The Institutional Review Board at Vanderbilt University approved all study methods and materials. Parents of all infant participants provided written informed consent prior to their participation in each visit of this study.

### Participants

Infants who participated in all three visits of the current study and completed a minimum of 10 sessions of parent-implemented training using either ‘sticky mittens’ or ‘non-sticky mittens’ (hereafter referred to as active training or passive training) were included in analyses. Half of the infants were randomly assigned to the active training condition, and the other half was assigned to the passive training condition. Infants completed three visits to the lab when they were approximately 3, 3.5, and 5.5 months of age. Participant characteristics did not differ between the two conditions (see **Table 1**). Data were collected from an additional 14 infants but were excluded from analyses because these infants became fussy during the first visit and were unable to complete the study session.

**Table 1:** Participant characteristics.

Condition	*N*	Race	Age V1	Age V2	Age V3
Active Training	16	W = 14	2 m, 20 d	3 m, 7 d	5 m, 10 d
	(females = 9)	A/W = 1	(*SD* = 6 d)	(*SD* = 10 d)	(*SD* = 11 d)
		B = 1			
Passive Training	16	A = 1	2 m, 21 d	3 m, 9 d	5 m, 10 d
	(females = 7)	W = 14	(*SD* = 7 d)	(*SD* = 5 d)	(*SD* = 8 d)
		B = 1			


*The total number of participants in each group (N) with the number of females per group specified in parentheses is reported in the second column. The number of infants whose parents reported their race as white (W), Asian (A), and black (B) are reported in the third column. Infants’ mean ages at visit one (V1), visit two (V2), and visit three (V3) are reported in months (m) and days (d), and the standard deviations are reported in parentheses in columns four through six.*

### Training

We designed our training conditions to provide similar experiences in terms of postural experience, exposure to the stimuli, and engaging in social interactions or sharing joint attention with caregivers. Our goal was to determine whether actively moving the toys, in comparison to passively watching a parent move the toys, benefitted infants’ object exploration skills. At the first lab visit, all parents were given toys, mittens, and received one-on-one demonstrations and verbal instructions about how to complete either active or passive mittens training with their infants. They also received printed directions describing the training procedure at the conclusion of the first visit. Parents were told that each training session should last approximately 10–12 min, or as long as infants were willing to participate. Parents recorded the dates and durations of training sessions on a log that they returned (along with the toys and mittens) at the second visit to the laboratory

#### Active Training

Parents first placed mittens on their infants’ hands. The palms of the mittens that babies wore in the active training condition were covered in strips of soft Velcro loop. Infants were seated either on adults’ laps or in a supported seat (such as a high chair or a well-anchored Bumbo seat) with their arms comfortably resting on a tabletop. Parents were instructed to present one toy at a time to their infants. The toys that infants interacted with in the active training condition were covered in strips of Velcro hook. When infants touched their mittened hands to these toys, the toys stuck to their mittens. Infants were thus able to lift the toys and move them through their visual fields. Each toy was placed within infants’ reach. Parents were told to verbally encourage their infants to contact the toys. If infants’ contacted the toys, parents removed the toys from the mittens after approximately 10 s.

#### Passive Training

As in the active training condition, parents first placed mittens on their infants’ hands. Unlike in the active training condition, however, the mittens that infants wore in the passive training condition did not have Velcro on them. Instead, the palms of infants’ mittens in passive training condition had strips of white ribbons sewn onto them to mimic the appearance of the white strips of Velcro. Likewise, the toys that infants saw in the passive training condition looked very similar to the toys infants interacted with in the active training condition, but the toys used in the passive training condition did not have any Velcro on them. Instead, these toys had strips of electrical tape on them to mimic the appearance of the black strips of Velcro on the toys babies interacted with in the active training condition. The passive training procedure required that parents move the toys to provide a visual experience for their infants that was similar to what infants experienced in active training. Parents were told to move the toys in a semi-circle around their infants’ bodies alternating between holding the toy at infants’ eye level and on the tabletop, tap the toys on the table to produce sounds, and touch the toys to the palms of their infants’ mittened hands. As in the active training condition, infants in the passive training condition only interacted with one toy at a time.

### Procedure

All infants included in the final sample completed three visits to our laboratory. The first visit consisted of an object exploration assessment followed by either active or passive mittens training. Between the first and second visits of this study, parents and infants trained at home using the mittens and toys we supplied. Infants returned to the laboratory for their second visit approximately 2 weeks after their first visit. At the second visit, infants participated in the object exploration assessment again, as well as a reaching assessment. The third visit took place approximately 2 months after the second visit. The third visit consisted of the same two measures infants completed at the second visit.

### Measures

#### Object Exploration Assessment

At each of the three visits, infants were presented with a red teether toy (Super Yummy Teether by Discovery Toys) for 30 s. The experimenter attempted to place the teether in infants’ hands. If infants dropped the teether before 15 s elapsed the experimenter replaced the teether in the infants’ hands. If more than 15 s had elapsed, the experimenter held the teether at the infants’ midlines until the conclusion of the assessment. If infants resisted grasping the teether, the experimenter held the teether at infants’ midlines throughout the duration of the assessment.

The durations of infants’ behaviors were coded by trained observers using frame-by-frame coding software (Libertus, 2008). The following behaviors were assessed: looking (gaze directed toward the teether), touching (manual contact with the teether), reaching (movement of the hand toward the teether), and bimanual exploration (touching the toy with both hands). Two observers coded all of the trials from the three visits using frame-by-frame coding software and overall reliability was high for looking (*ICC* = 0.88), touching (*ICC* = 0.99), reaching (*ICC* = 0.86), and bimanual exploration (*ICC* = 0.98).

#### Reaching Assessment

At the second and third visits, the experimenter presented infants with a rattle (Sassy Flip and Grip) to assess their reaching skills. During this 2 min assessment, the rattle was moved incrementally closer to infants. In total, the rattle was placed in four positions: out of infants’ reach, within infants’ reach but far from the infant, within infants’ reach and close to them, and in infants’ hands. The experimenter placed the rattle in each of these positions for 30 s. Each time she placed the rattle into a new position, the experimenter called the infant’s attention to the rattle by looking at it, pointing toward it, and enthusiastically exclaiming “Look! What is this? Do you want this? Can you get it?”

Just as in the assessment of object exploration, the durations of infants’ behaviors during the reaching assessment were coded by trained observers using frame-by-frame coding software (Libertus, 2008). Trained research assistants coded for looking, touching, reaching, and bimanual exploration behaviors just like in the assessment of object exploration. Additionally, these research assistants coded infants’ grasping (fingers encircling or gripping the toy) during the phase of the reaching assessment when the rattle was placed in the infants’ hands. Two observers coded a random sample of trials from each visit using frame-by-frame coding software, and overall reliability was high for looking (*ICC* = 0.91), touching (*ICC* = 0.96), reaching (*ICC* = 0.85), bimanual exploration (*ICC* = 0.90), and grasping (*ICC* = 0.99).

### Analysis

Two repeated measures multivariate analyses of variance (MANOVAs) were used to assess changes in infants’ behaviors between visits 1 and 2, and visits 1 and 3 during the assessment of object exploration. Visit 1 served as a baseline measure of infants’ initial exploration durations, allowing us to measure changes in their behaviors over time. Separate MANOVAS were performed because we hypothesized that bimanual exploration would be minimal at visits 1 and 2 and would increase by visit 3. Thus, our model for testing changes in infants’ behaviors from visit 1 to visit 2 measured changes in looking, touching, and reaching, and our model for testing changes in behaviors from visit 1 to visit 3 included looking, touching, reaching, and bimanual exploration.

Repeated measures MANOVAs were also used to assess changes in infants’ behaviors between visit 2 and visit 3 during the three phases (out of reach, within reach, and in hand) of the reaching assessment. Since we were testing for changes in different dependent variables during each phase of this assessment, we used three separate repeated measures MANOVAS. Partial eta-squared ($\eta_{p}^{2}$), a measure of effect size, is reported for all MANOVAs. *T*-tests were performed for follow-up analyses. Difference scores were calculated by subtracting the duration of infants’ behaviors at earlier visits from the durations of their behaviors at later visits. Cohen’s d (*d*) was calculated for all follow up analyses.

## Results

### Object Exploration Assessment

We used a repeated measures MANOVA to assess potential differences in infants’ exploration behaviors in each condition at visit 1 and visit 2. Visit (first or second) was entered as a within-subject factor, and condition (active or passive) was entered as a between-subject factor. Three dependent variables, all of which were durations of exploration behaviors, were tested: looking, touching, and reaching.

The analysis comparing visits 1 and 2 showed no significant differences.

A second analysis assessed changes from visit 1 to visit 3 in four dependent variables: looking, touching, reaching, and bimanual exploration. Visit (first or third) was entered as a within-subject factor, and condition (active or passive) was entered as a between-subject factor.

This MANOVA revealed a main effect of visit on infant’ looking behaviors, *F*(1,29) = 6.08, *p* = 0.020, $\eta_{p}^{2}$ = 0.173. Across both groups, on average infants looked less at the teether at visit 3 (*M*_V 3_ = 14.17, *SD*_V 3_ = 8.22) compared to visit 1 (*M*_V 1_ = 18.85, *SD*_V 1_ = 10.72). The main effect of condition was non-significant [*F*(1,29) = 0.071, *p* = 0.792, $\eta_{p}^{2}$ = 0.002], but there was a significant interaction between visit and condition, *F*(1,29) = 9.88, *p* = 0.004, $\eta_{p}^{2}$ = 0.254. Planned comparisons revealed that infants who participated in passive training (*M*_V 1_ = 21.71, *SD*_V 1_ = 8.15, *M*_V 3_ = 10.60, *SD*_V 3_ = 7.2), significantly decreased their looking toward the teether from visit 1 to visit 3, *t*(14) = -3.69, *p* = 0.002, 95% *CI* [-17.57, -4.65], *d* = -1.97. In contrast, infants in the active training condition (*M*_V 1_ = 16.18, *SD*_V 1_ = 12.32, *M*_V 3_ = 17.52, *SD*_V 3_ = 7.86) maintained the same amount of looking toward the teether from visit 1 to visit 3, *t*(15) = 0.52, *p* = 0.613, 95 % *CI* [-4.19, 6.87], *d* = 0.27.

This MANOVA also revealed a significant main effect of visit on the durations of infants’ touching behaviors, *F*(1,29) = 199.61, *p* < 0.001, $\eta_{p}^{2}$ = 0.873. Across both training conditions, infants tended to touch the teether more at visit 3 (*M*_V 3_ = 22.54, *SD*_V 3_ = 8.15) than at visit 1 (*M*_V 1_ = 3.02, *SD*_V 1_ = 4.84). The main effect of condition was non-significant [*F*(1,29) = 0.055, *p* = 0.816, $\eta_{p}^{2}$ = 0.002], but again we found a significant interaction between visit and condition, *F*(1,29) = 7.96, *p* = 0.009, $\eta_{p}^{2}$ = 0.215. Planned comparisons showed that infants in the active training condition [*M*_V 1_ = 1.36, *SD*_V 1_ = 3.17, *M*_V 3_ = 24.63, *SD*_V 3_ = 5.92, *t*(15) = 15.04, *p* < 0.001, 95% *CI* [19.97, 26.57], *d* = 4.82] as well as the passive training condition [*M*_V 1_ = 4.78, *SD*_V 1_ = 5.75, *M*_V3_ = 20.31, *SD*_V3_ = 9.73, *t*(14) = 6.72, *p* < 0.001, 95% *CI* [10.57, 20.47], *d* = 1.85] increased their durations of touching the teether from visit 1 to visit 3. However, this increase in durations of touching from visit 1 to visit 3 was larger among infants in the active condition compared to the passive condition.

The main effect of visit on infants’ reaching behaviors was non-significant, *F*(1,29) = 0.031, *p* = 0.861, $\eta_{p}^{2}$ = 0.001. We did find a significant main effect of condition on reaching behaviors, *F*(1,29) = 7.39, *p* = 0.011, $\eta_{p}^{2}$ = 0.237. Across both visits, infants in the passive condition (*M* = 4.56, *SD* = 4.87) spent more time reaching for the teether than infants in the active condition (*M* = 2.06, *SD* = 2.55). The interaction between visit and condition was non-significant, *F*(1,29) = 1.78, *p* = 0.193, $\eta_{p}^{2}$ = 0.058. In light of our pattern of findings that infants in the active condition increased the amount of time they spent touching the teether from visit 1 to visit 3 more than infants in the passive condition, we interpret these reaching findings to show that infants in the passive condition spent more time struggling to attain the teether at visit 3 whereas infants in the active condition appear to have more successfully maintained manual contact with the teether.

Lastly, we found a significant main effect of visit on infants’ bimanual behaviors, *F*(1,29) = 49.24, *p* < 0.001, $\eta_{p}^{2}$ = 0.629. Across conditions, infants tended to engage in more bimanual actions at visit 3 (*M*_V 3_ = 11.35, *SD*_V 3_ = 10.07) as compared to visit 1 (*M*_V 1_ = 0.03, *SD*_V 1_ = 0.14). We also found a significant main effect of condition on infants’ bimanual behaviors [*F*(1,29) = 9.00, *p* = 0.005, $\eta_{p}^{2}$ = 0.237], with infants in the active condition engaging in greater durations of bimanual behaviors across visits 1 and 3 (*M* = 7.97, *SD* = 10.61) than infants in the passive training condition (*M* = 3.25, *SD* = 6.52). The interaction between visit and condition was significant as well, *F*(1,29) = 9.12, *p* = 0.005, $\eta_{p}^{2}$ = 0.239. Planned comparisons showed that infants in the active condition [*M*_V 1_ = 0.00, *SD*_V 1_ = 0.00, *M*_V 3_ = 15.94, *SD*_V 3_ = 9.85_,_ *t*(15) = 6.47, *p* < 0.001, 95% *CI* [10.69, 21.19], *d* = 2.29] as well as the passive condition [*M*_V 1_ = 0_._05, *SD*_V 1_ = 0.21, *M*_V 3_ = 6.45, *SD*_V 3_ = 7.97, *t*(14) = 3.09, *p* = 0.008, 95% *CI* [1.95, 10.83], *d* = 1.25] increased their durations of bimanual engagement from visit 1 to visit 3, but this increase was greater among infants in the active condition. In short, infants engaged in very little bimanual actions at visit 1, but quite a lot at visit 3. At visit 3 the infants in the active training condition outperformed infants in the passive training condition in terms of performing greater durations of bimanual actions.

### Reaching Assessment

The reaching assessment was analyzed in three parts: Looking and reaching were assessed when the rattle was out of infants’ reach; looking, reaching, grasping, touching, and bimanual exploration were all assessed during the combined portions of the assessment where the rattle was within infants’ reach; looking, grasping, touching, and bimanual exploration were assessed during the portion of the assessment when the rattle was placed in infants’ hands.

During phase 1, the rattle was purposely placed outside of infants’ reach for 30 s. Thus, we did not analyze infants’ touching, grasping, or bimanual actions during this phase of the reaching assessment. Rather, we limited our analyses of the first phase to looking and reaching behaviors.

In terms of infants’ looking behaviors, the main effect of visit was non-significant, *F*(1,30) = 0.03, *p* = 0.873, $\eta_{p}^{2}$ = 0.001. We did find a significant main effect of condition, *F*(1,30) = 21.64, *p* < 0.001, $\eta_{p}^{2}$ = 0.419. Across visits 2 and 3, infants in the active condition (*M* = 14.73, *SD* = 9.06) tended to look more at the rattle during phase 1 when the rattle was outside of their reach compared to the passive condition (*M* = 7.08, *SD* = 5.90). The interaction between visit and condition was non-significant, *F*(1,30) = 0.25, *p* = 0.618, $\eta_{p}^{2}$ = 0.008.

The main effect of visit on infants’ durations of reaching was significant, *F*(1,30) = 2.98, *p* = 0.095, $\eta_{p}^{2}$ = 0.090. Across both training conditions, infants reaching behaviors increased from visit 2 (*M* = 2.57, *SD* = 3.58) to visit 3 when the rattle was placed out of reach. The main effect of condition [*F*(1,30) = 0.048, *p* = 0.828, $\eta_{p}^{2}$ = 0.002], and the interaction between visit and condition were both non-significant, *F*(1,30) = 0.04, *p* = 0.836, $\eta_{p}^{2}$ = 0.001.

During the second phase of the reaching assessment, the rattle was placed in two positions within infants’ reach. We combined these two 30-s phases of this assessment, and we used another repeated measures MANOVA to analyze the changes in durations of infants’ looking, touching, reaching, and grasping behaviors during this portion of the assessment from visit 2 to visit 3.

This MANOVA showed a marginally significant main effect of visit on infants’ looking durations, *F*(1,30) = 4.03, *p* = 0.054, $\eta_{p}^{2}$ = 0.118. At visit 2 (*M*_V 2_ = 31.29, *SD*_V 2_ = 15.09) infants tended to look less at the rattle compared to during visit 3 (*M*_V 3_ = 38.17, *SD*_V 3_ = 13.26). There was a significant main effect of condition on infants’ looking behaviors, *F*(1,30) = 10.48, *p* = 0.003, $\eta_{p}^{2}$ = 0.259. Averaged across both visits, infants in the active condition (*M* = 39.91, *SD* = 14.99) tended to look more at the rattle during the second phase of the reaching assessment compared to infants in the passive training condition (*M* = 29.5, *SD* = 12.12). The interaction between visit and condition was non-significant, *F*(1,30) = 0.65, *p* = 0.428, $\eta_{p}^{2}$ = 0.021.

The main effect of visit on infants’ durations of touching was significant, *F*(1,30) = 64.43, *p* < 0.001, $\eta_{p}^{2}$ = 0.682. Across both conditions, infants tended to touch the rattle more when it was within reach at visit 3 (*M*_V 3_ = 30.75, *SD*_V 3_ = 15.24) as compared to visit 2 (*M*_V 2_ = 8.28, *SD*_V 2_ = 7.99). The main effect of condition was also significant, *F*(1,30) = 5.58, *p* = 0.025, $\eta_{p}^{2}$ = 0.157. Infants in the active training condition (*M* = 23.03, *SD* = 19.19) tended to touch the rattle for greater durations across the two visits compared to infants in the passive training condition (*M* = 16.00, *SD* = 12.77). The interaction between visit and condition was marginally significant, *F*(1,30) = 3.08, *p* = 0.089, $\eta_{p}^{2}$ = 0.093. Infants in both the active [*M*_V 2_ = 9.34, *SD*_V 2_ = 7.57, *M*_V 3_ = 36.73, *SD*_V 3_ = 17.35, *t*(15) = 5.94, *p* < 0.001, 95% CI [17.56, 37.22], *d* = 2.01] and passive training conditions [*M*_V 2_ = 7.23, *SD*_V 2_ = 8.34, *M*_V 3_ = 24.78, *SD*_V 3_ = 10.16, *t*(15) = 5.53, *p* < 0.001, 95% CI [10.79, 24.33], *d* = 1.89] increased their durations of touching behaviors from the first to the second visit, with this pattern being more pronounced among the infants with active training.

This MANOVA revealed a significant main effect of visit on infants’ durations of reaching, *F*(1,30) = 8.45, *p* = 0.007, $\eta_{p}^{2}$ = 0.220. Infants, overall, tended to reach more at the second visit (*M*_V 2_ = 8.93, *SD*_V 2_ = 8.55) as opposed to the third visit (*M*_V 3_ = 4.22, *SD*_V 3_ = 3.77). Infants may have experienced greater success in attaining the rattle during this portion of the reaching assessment at visit 3 compared to at visit 2. The main effect of condition [*F*(1,30) = 1.10, *p* = 0.303, $\eta_{p}^{2}$ = 0.035] and the interaction between visit and condition were non-significant [*F*(1,30) = 1.17, *p* = 0.289, $\eta_{p}^{2}$ = 0.037].

The fourth dependent variable analyzed by this MANOVA was durations of infants’ grasping behaviors from visit 2 to visit 3. There was a significant main effect of visit, *F*(1,30) = 69.33, *p* < 0.001, $\eta_{p}^{2}$ = 0.698. As a whole, infants grasped the rattle much more at visit 3 (*M*_V 3_ = 24.81, *SD*_V 3_ = 15.39) than at visit 2 (*M*_V 2_ = 2.71, *SD*_V 2_ = 5.69). The main effect of condition was also significant, *F*(1,30) = 9.57, *p* = 0.004, $\eta_{p}^{2}$ = 0.242. Across the two visits, infants in the active condition (*M* = 17.80, *SD* = 19.17) tended to engage in greater durations of grasping than infants in the passive condition (*M* = 9.71, *SD* = 10.95). Finally, we found a significant interaction between visit and condition, *F*(1,30) = 5.56, *p* = 0.025, $\eta_{p}^{2}$ = 0.156. Planned comparisons revealed that infants in the active condition [*M*_V 2_ = 3.63, *SD*_V 2_ = 7.56, *M*_V 3_ = 31.98, *SD*_V 3_ = 16.53, *t*(15) = 6.14, *p* < 0.001, 95% *CI* [18.51, 38.21], *d* = 2.22], as well as the passive condition [*M*_V 2_ = 1.79, *SD*_V 2_ = 2.82, *M*_V 3_ = 17.63, *SD*_V 3_ = 10.30, *t*(15) = 6.07, *p* = < 0.001, 95% *CI* [10.28, 21.40], *d* = 2.04] increased their durations of grasping from visit 2 to visit 3, but this increase was significantly greater among infants in the active training condition.

A third repeated measures MANOVA analyzed infants’ exploration behaviors during the third portion of the reaching assessment, when the rattle was placed in infants’ grasp. Looking, touching, grasping, and bimanual actions were entered as dependent variables in this MANOVA. We did not include reaching as a dependent variable because for the most part, the rattle very close to or held in infants’ hands.

We found a marginally significant main effect of visit on infants’ looking behaviors, *F*(1,27) = 3.01, *p* = 0.094, $\eta_{p}^{2}$ = 0.100. Across both conditions, infants looked more at visit 3 (*M*_V 3_ = 18.63, *SD*_V 3_ = 8.50) than at visit 2 *(M*_V 2_ = 14.11, *SD*_V 2_ = 11.77). The main effect of condition was non-significant, *F*(1,27) = 2.54, *p* = 0.123, $\eta_{p}^{2}$ = 0.086, but the interaction between visit and condition was marginally significant, *F*(1,27) = 3.05, *p* = 0.092, $\eta_{p}^{2}$ = 0.101. Infants in the active condition significantly increased their durations of looking from visit 2 (*M*_V 2_ = 13.87, *SD*_V 2_ = 11.04) to visit 3 [*M*_V 3_ = 22.64, *SD*_V 3_ = 8.09), *t*(14) = 2.99, *p* = 0.010, 95% *CI* [2.48, 15.06], *d* = 0.89]. In comparison, infants in the passive training condition maintained nearly identical durations of looking from visit 2 (*M*_V 2_ = 14.36, *SD*_V 2_ = 12.92) to visit 3 [*M*_V3_ = 14.34, *SD*_V3_ = 6.84, *t*(13) = -0.01, *p* = 0.994, 95% *CI* [-9.02,8.96], *d* = -0.003].

The main effects of visit [*F*(1,27) = 1.62, *p* = 0.215, $\eta_{p}^{2}$ = 0.056], condition [*F*(1,27) = 1.98, *p* = 0.171, $\eta_{p}^{2}$ = 0.068], and the interaction between visit and condition [*F*(1,27) = 0.20, *p* = 0.659, $\eta_{p}^{2}$ = 0.007] for touching behaviors were all non-significant. Similarly, the main effects of visit [*F*(1,27) = 0.73, *p* = 0.400, $\eta_{p}^{2}$ = 0.026], condition [*F*(1,27) = 2.54, *p* = 0.123, $\eta_{p}^{2}$ = 0.086], and the interaction between visit and condition [*F*(1,27) = 0.77, *p* = 0.287, $\eta_{p}^{2}$ = 0.028] were non-significant for infants’ grasping behaviors.

Finally, we found a main effect of visit on infants’ bimanual exploration behaviors, *F*(1,27) = 8.12, *p* = 0.008, $\eta_{p}^{2}$ = 0.231. Infants, across the active and passive training conditions, performed longer durations of bimanual exploration at the third visit (*M*_V 3_ = 12.49, *SD*_V 3_ = 9.95) compared to the second visit (*M*_V 2_ = 5.19, *SD*_V 2_ = 8.19). The main effect of condition [*F*(1,27) = 0.38, *p* = 0.543, $\eta_{p}^{2}$ = 0.014] and the interaction between visit and condition were both non-significant, *F*(1,27) = 0.09, *p* = 0.771, $\eta_{p}^{2}$ = 0.003.

## Discussion

The current study examined how active and passive mittens training affected infants’ exploration behaviors immediately following and 2 months after 2 weeks of parent-led training sessions. Recent findings provide evidence that 2 weeks of active mittens training positively affected infants’ visual and manual engagement with a wooden tabletop bead maze toy and parents’ ratings of their children’s attention spans using the Early Childhood Behavior Questionnaire (ECBQ; Putnam et al., 2006) 12 months after training concluded (Libertus et al., in press). The current study helps to illuminate how mittens training influences infants’ motor skills during the interim period, 2 months after the conclusion of training. Because the measures in the current study were designed for 5-month-old infants and the measures in the Libertus et al. (in press) study were designed for 15-month-old infants, these two sets of results cannot be directly compared. Despite this fact, the findings from these two studies are quite consistent with each other. Both studies show evidence of increased visual and manual contact with objects for infants who participated in active mittens experience, compared to those who participated in passive mittens experience. The findings from the current study also indicate that the effect of active mittens training increases between the second and third visits, consistent with a cascading or ‘snowballing’ effect.

In contrast to our expectations and our prior findings, we did not find differences in infants’ exploration patterns between the two training groups immediately after training concluded. We believe this lack of an effect stemmed from a small difference in the procedure we used to conduct the object exploration task in the immediate post-training session. Specifically, the object exploration trial was only 30 s long instead of 60 s long as it was in our prior research. Our attempt to streamline the procedure may have diminished the differences between the training conditions on this measure.

**FIGURE 1:**
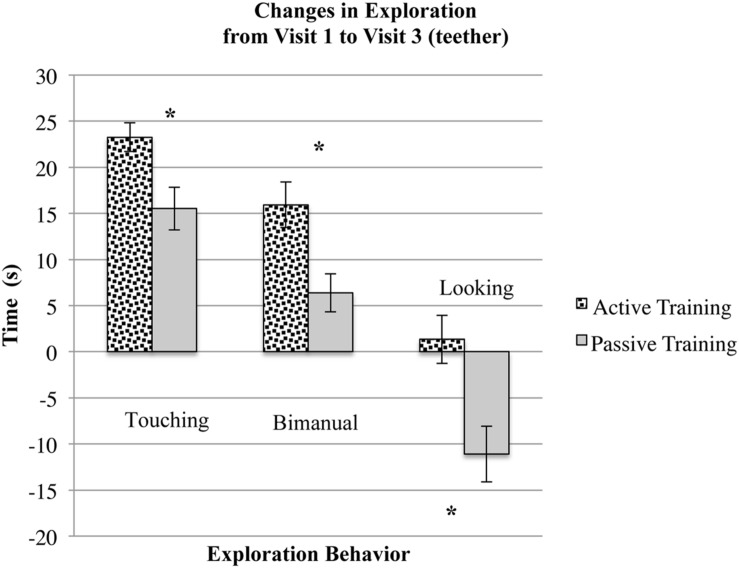
**Infants who participated in active mittens training increased their durations of touching and bimanually exploring the teether between visit 1 and visit 3 significantly more than their peers who participated in passive mittens training at 3 months of age.** Infants who participated in passive training engaged in similar amounts of looking toward the teether at visits 1 and 3, while infants who participated in passive training looked significantly less at the teether during visit 3. Asterisks indicate significant differences (*p* < 0.05).

Two months after the end of training, however, we found several differences in infants’ exploration behaviors, with longer durations of exploration behaviors among infants who had participated in active as opposed to passive mittens training (See **Figure 1**). This pattern of findings supports the idea of a developmental cascade because the effects of mittens training became more substantial over time. First, in our object exploration assessment, infants who participated in active mittens training showed significantly larger increases in looking and bimanual exploration, and they showed different patterns of reaching between visits one and three as compared to their peers who participated in passive mittens training.

**FIGURE 2:**
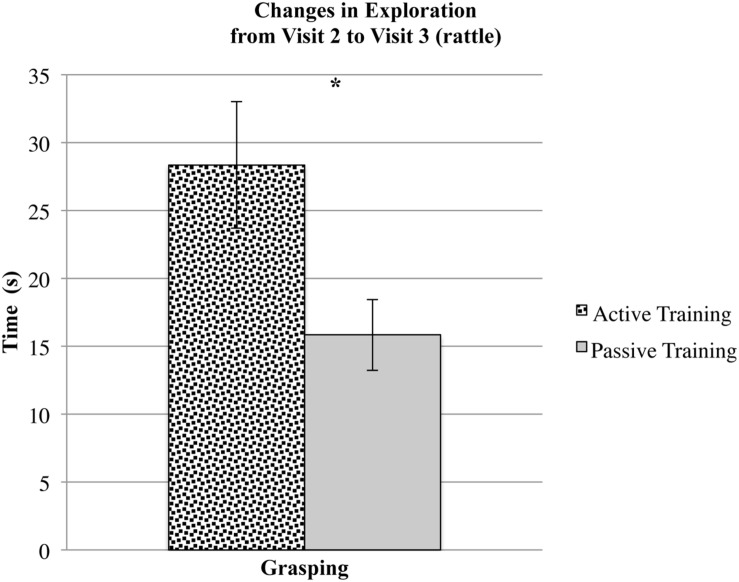
**All infants grasped the rattle for significantly longer at visit 3 compared to visit 2, but this increase was larger for infants who participated in active training compared to infants who participated in passive training.** Asterisks indicate significant differences (*p* < 0.05).

Second, infants who participated in active mittens training 2 months earlier showed significantly greater increases in grasping (see **Figure 2**) and marginally significant increases in touching and looking behaviors in this assessment. Together, these results indicate that active mittens training positively affected infants’ object exploration skills and their motivation to explore. Although these differences between the groups were not noticeable immediately after training, the benefits of active mittens training became more substantial over time.

### Object Exploration

These results are consistent with the idea that boosting infants’ object exploration skills has cascading effects across development. One reason we make this conclusion is that the differences between our active and passive training groups became more pronounced over time. This pattern of results is consistent with the idea that the infants’ abilities continue to build over time. A second reason we consider a developmental cascade a good model for our findings is that the active training procedure did not ‘train up’ the specific actions we coded in our testing assessments. Because there is not an obvious causal connection between the training procedures and the test measures that improved after training, it suggests to us that there could be some intervening factors that we do not yet know about.

For example, our results indicate that active mittens training leads to increases in infants’ bimanual object exploration. Bimanual exploration is initially used as infants transport objects to their mouths for oral exploration. Oral exploration can help infants to glean information about object properties such as texture, shape, and affordances (Rochat, 1987). More experience detecting objects’ affordances may increase infants’ awareness of the properties of objects. Later, bimanual exploration is linked with infants’ role differentiated bimanual manipulations in which they use one hand to stabilize the object and their other hand to act on the object (Rochat, 1989; Nelson et al., 2013). Using two hands to explore an object may allow infants to engage in more kinds of actions on the object and garner more information about the object than they can when using only one hand. The development of sophisticated exploration techniques expands learning opportunities available to infants. Object exploration skills may also be linked to infants’ perceptions of their surroundings. Baumgartner and Oakes (2013) found that infants who showed more advanced exploratory behaviors were more successful in detecting changes in object properties during a dynamic event. More experience detecting objects’ affordances should increase infants’ awareness of the properties of objects.

Object exploration skills may serve as a foundation for later skills. Many longitudinal studies have shown that motor development during infancy can be predictive of later outcomes (Bornstein et al., 2013; Bornstein, 2014). Research suggests that babies with more sophisticated motor skills during early infancy might have increased attention spans (Friedman et al., 2005) and engage in more advanced symbolic play as young children (Tamis-LeMonda and Bornstein, 1993). Motor development at 5 months has been linked to academic outcomes among teenagers (Bornstein et al., 2013). Based on these findings, one might expect that sticky mittens training, by boosting 5-month-old infants’ object exploration skills, might have cascading effects across development. Indeed, there are other recently reported findings that provide evidence of differences in 15-month-old infants’ object engagement depending upon whether they had received active or passive mittens training 1 year earlier (Libertus et al., in press).

### Implications

During active mittens training, parents spent a great deal of time encouraging their infants to make contact with objects and cheering them on when they were successful in doing so. It is likely that this parent–child experience contributed to the benefits in infants’ motor skills evidenced in the current study. Interventions that are confined to laboratories or clinics might not have as large of an impact on infants’ behaviors because they are not imbedded in infants’ daily lives and familial relationships. Research shows that when parents are able to incorporate early motor interventions into daily activities with their infants, outcomes tend to be more positive (Mahoney and Perales, 2006). A review by Hadders-Algra (2013) reiterates this point by recommending that parents need to be involved in offering opportunities for successful reaching among children who are at risk for reaching delays. For example, they suggest that parents ensure their babies have adequate postural support by using cushions or holding their babies’ midsections to make reaching easier for them. Parents spend a great deal of time with their infants, and they therefore are capable of (a) implementing interventions in a wider variety of contexts and settings, and (b) scaffolding their infants’ experiences in these settings, both of which would be helpful in promoting infants’ learning.

Active mittens training may prove to be a successful intervention for infants who are at risk for reaching delays. For example, promising findings suggest that infants who are at risk for developing autism showed increased grasping behaviors after participating in active mittens training (Libertus and Landa, 2014). It is yet to be explored whether infants at risk reaching delays due to factors such as prematurity or Down syndrome might also benefit from this early motor intervention. Fortunately, active mittens training is inexpensive, the training materials can be easily transported, and short training sessions (10–20 min a day) are recommended. For these reasons, the parents of infants who are at risk for reaching delays might find this early motor intervention appealing.

### Limitations

Several limitations in the design and interpretation of this study should be acknowledged. The participants in this study are mostly of Caucasian ethnicity and members of a high socio-economic group (as measured by parental education and occupation). Our findings are also based on a small sample size. Additionally, infants whose parents were unable to complete the minimum amount of training and infants who did not return for each of the three laboratory visits were excluded from analyses.

All infants who participated in our study took part in an at-home training regimen. It could therefore be argued that this study does not include a true control condition. However, past research with sticky mittens has compared infants with active training to infants with no training experience (Needham et al., 2002). This study found that infants with active mittens training showed greater object exploration skills compared to their peers without training.

We did not take measures to ensure that parents adhered to training protocol. Parents were responsible for completing a log detailing the frequency and duration of mittens training sessions with their infants. While we are unable to confirm that parents were honest in reporting their training or that they strictly followed the directions we provided, our results indicate that the experiences of infants in the two training conditions led to differing exploration skills. Thus, we feel confident that parents indeed followed training protocol.

While the findings of this study may appear to conflict with prior published research showing immediate benefits of active mittens training once training concludes (Needham et al., 2002; Libertus and Needham, 2010; Libertus and Landa, 2014), we think that this discrepancy is most likely because of subtle differences in our pre- and post-training assessments. Needham et al. (2002) used an identical red teether during pre- and post-training assessments, but infants were given the opportunity to explore the teether for 1 min during four separate trials. In the current study, infants were only permitted to explore the teether for 30 s during pre- and post-training assessments. This shorter duration of measures may have prevented us from detecting significant differences in the exploration behaviors among our training conditions. Similarly, Libertus and Needham (2010) found significant differences among active and passive mittens training conditions, but pre- and post-training assessments were each 2 min long, whereas in the current study infants’ pre- and post-training assessments were much shorter. Infants in Libertus and Needham (2010) were also reaching for a rattle that was moved closer to them during four sequential phases of the pre- and post-training assessments rather than exploring a teether that was placed in their grasps. Libertus and Landa (2014) used an abbreviated, 1-min long, version of the pre- and post-training assessments with infants reaching for a rattle used in Libertus and Needham (2010). Procedural differences in pre- and post-training assessments may therefore help to explain this apparent inconsistency in findings between prior studies and the current study.

## Conclusion

The results of the current study suggest that active mittens training provides an opportunity for pre-reaching infants to actively engage with objects through reaching and grasping, thus facilitating their early motor development. Two months after the conclusion of this early motor intervention, infants who participated in active mittens training showed increased object exploration skills, engaging in complex object engagement patterns such as bilateral exploration, compared to their peers who participated in passive mittens training. We interpret these findings as evidence that early motor experience through active mittens training motivates infants to begin practicing their reaching and grasping, which leads to improvements in their object exploration skills. By drawing parents’ attention to their infants’ motor achievements, it is likely that parents may encourage their infants’ efforts and provide more opportunities for their infants to practice their new motor skills. Future research will examine how such increases in object exploration skills relate to later outcomes, such as language development, tool use, and attention span.

## Author Contributions

SW analyzed the data and wrote the manuscript. RW completed data collection and did preliminary analyses and helped write the manuscript. AWN designed the study, oversaw data collection, contributed to writing the manuscript, and provided funding for this research.

## Conflict of Interest Statement

The authors declare that the research was conducted in the absence of any commercial or financial relationships that could be construed as a potential conflict of interest.
